# Tumor Suppressor p53 Functions as a Negative Regulator in IgE-Mediated Mast Cell Activation

**DOI:** 10.1371/journal.pone.0025412

**Published:** 2011-09-23

**Authors:** Kotaro Suzuki, Samantha H. Murphy, Yifeng Xia, Masaya Yokota, Daiki Nakagomi, Fei Liu, Inder M. Verma, Hiroshi Nakajima

**Affiliations:** 1 Laboratory of Genetics, Salk Institute for Biological Studies, La Jolla, California, United States of America; 2 Department of Molecular Genetics, Graduate School of Medicine, Chiba University, Chiba, Japan; Oklahoma Medical Research Foundation, United States of America

## Abstract

Mast cells are known to play a pivotal role in allergic diseases such as allergic rhinitis, asthma, and atopic dermatitis by releasing granules containing histamine, LTC4, and other preformed chemical mediators. Previous reports have demonstrated that IKK2 (also called IKKβ), a central intracellular component of NF-κB activation pathways, plays a critical role in IgE-mediated degranulation of mast cells and anaphylaxis in mice. In this study, we show that protein levels of tumor suppressor p53 are up-regulated upon IgE-mediated activation in mast cells and lack of p53 results in enhanced responses in both early and late phase anaphylaxis. p53 inhibits not only the catalytic activity of IKK2 presumably through the modulation of glycosylation but also p65 (RelA)-mediated transactivation. Our findings are the first to demonstrate that p53 functions as a negative regulator in mast cells.

## Introduction

Mast cells are recognized as the major effector cells of the type I hypersensitivity reactions and they are known to play a pivotal role in allergic diseases, such as allergic rhinitis, asthma, and atopic dermatitis. Engagement of FcεRI by IgE, followed by the aggregation of multiple IgE-bearing FcεRI molecules by polyvalent antigens, leads to degranulation and release of histamine, LTC4, and other preformed chemical mediators [1], [2]. Additionally, multiple cytokine genes are transcribed and newly synthesized arachidonic acid metabolites are secreted [1], [2].

It is well established that mast cells promote the early phase of type I hypersensitivity reactions by releasing granule contents after FcεRI-crosslinking. Recently, we have demonstrated that IKK2, which is a central component of the intracellular signaling pathway mediating NF-κB activation [3]–[6], plays critical roles in IgE-mediated anaphylaxis in vivo and IgE-mediated degranulation of mast cells in vitro [7]. Upon FcεRI stimulation, IKK2 phosphorylates SNAP-23, the target membrane soluble N-ethylmaleimide-sensitive fusion factor attachment protein receptor (SNARE), leading to degranulation and anaphylactic reactions [7]. Moreover, accumulating evidence has shown that the kinase activity of IKK2 is regulated by post-translational modifications [6]. However, it is still unknown whether the post-translational modifications of IKK2 are involved in IKK2-mediated degranulation of mast cells and anaphylactic reactions.

Transcription factors of the NF-κB family, which consists of NF-κB1, NF-κB2, p65 (also called RelA), c-Rel, and RelB, regulate the expression of hundreds of genes in the context of multiple important biological processes, such as apoptosis, proliferation, innate and adaptive immune responses, and inflammation [3], [4]. p65 has been shown to play critical roles in IKK2-mediated gene induction of proinflammatory cytokines [3], [4]. It has also been shown that the activity of p65 is modulated by several other molecules including CREB binding protein, glucocorticoid receptor, and SP1 transcription factor [6]. However, the regulatory mechanisms underling p65-mediated gene induction of proinflammatory cytokines in mast cells are still largely unknown.

Tumor suppressor p53 is a sequence-specific transcription factor that is critical for maintaining genomic stability [8]. Without cellular stresses, protein levels of p53 in cells are maintained at low levels and the majority of p53 remains in the cytoplasm. Upon induction of various stresses such as apoptosis, cell cycle arrest, senescence, DNA repair, cell metabolism, and autophagy [9]–[12], the half-life of p53 increases from minutes to hours. p53 then translocates into the nucleus and activates its target genes. Although the roles of p53 in stress-associated stimulation have been well studied, the roles of p53 in antigen receptor-mediated stimulation are poorly understood.

In this study, we show that the protein levels of p53 are up-regulated upon IgE-mediated activation in mast cells and that the lack of p53 results in enhanced mast cell activation in vivo as well as in vitro. p53 inhibits not only the catalytic activity of IKK2 through the modulation of glycosylation but also p65-mediated transactivation. Our findings indicate that_p53 functions as a negative regulator of mast cell activation through the inhibition of NF-κB pathways.

## Methods

### Mice

p53-deficient (p53^−/−^) mice on a C57BL/6 background [13] were purchased from Taconic Farms (Hudson, NY). Mast cell-deficient WBB6F1-W/W^v^ mice (W/W^v^ mice) were purchased from SLC (Shizuoka, Japan). p65-heterozygous mice [14] were kindly provided by Dr. Baltimore (California Institute of Technology, USA). Mice were bred and housed in the animal care facility at Salk Institute and Chiba University. Experimental procedures involving mice followed the guidelines from the National Institutes of Health and Chiba University and were approved by the Animal Use Committee (approval ID; 09-052) at the Salk Institute and at Chiba University (approval ID; 21-11).

### Flow cytometric analysis

Cells were analyzed on FACSCalibur (Becton Dickinson, San Jose, CA) with CELLQuest software. The following antibodies were purchased: anti-CD117 (c-kit) allophycocyanin (APC) (2B8; BD Biosciences, San Diego, CA) and anti-FcεRIα (FITC) (MAR-1; eBioscience, San Diego, CA). Before staining, Fc receptors were blocked with anti-CD16/32 antibody (2.4G2; BD Biosciences). Negative controls consisted of isotype-matched directly conjugated, nonspecific antibodies (BD Biosciences).

### Histological quantification of mast cells

Peritoneal mast cells were stained with anti-CD117 (c-kit) APC and anti-FcεRIα (FITC) and analyzed by flow cytometry. The numbers of mast cells in the tissue were counted by light microscopy (x400) on Alcian blue-stained sections. Data were expressed as the number of mast cells per mm^2^ in ear dermis and the number of mast cells per villus crypt unit in jejunal mucosa.

### Cell culture

Primary culture of IL-3-dependent bone marrow-derived mast cells (BMMCs) was prepared from 6- to 8-week-old WT and p53^−/−^ mice and maintained as previously described [15]. Approximately 99% of cells recovered after 4 weeks of culture were morphologically mast cells.

### Reconstitution of mast cells in W/W^v^ mice with BMMCs

For reconstitution of skin mast cells in W/W^v^ mice, WT BMMCs or p53^−/−^ BMMCs (1×10^6^ cells in 20 µl of PBS) were injected intradermally into the right ear of W/W^v^ mice as described previously [7]. As controls, PBS was injected to the left ear.

### Passive cutaneous anaphylaxis

To induce passive cutaneous anaphylaxis (PCA), 4 weeks after the transplantation of BMMCs, the mice were sensitized with monoclonal mouse dinitrophenol-specific (anti-DNP) IgE (100 ng in 20 µl of PBS, Sigma, St Louis, MO) by intradermal injection into the ear. Twenty-four hours later, 200 µg of DNP-HSA (human serum albumin; Sigma) diluted in sterile saline were injected intravenously to the mice. Ear swelling was quantified by three consecutive measurements of ear thickness using calipers before (base line) and 1, 2, 3, 4, and 6 hours after the antigen challenge. In some experiments, 0.5% Evans blue dye was injected together with DNP-HSA. Where indicated, 2-deoxy-D-glucose (2-DG, 50 µl of 0.2 mM/mouse) were given intravenously to the mice at 20 hours before the antigen challenge.

### Measurement of Evans blue dye extravasation

To extract Evans blue dye from the tissue, ear biopsy specimens were incubated in 0.3 ml of formamide at 60°C for 4 days. Absorption was measured at 620 nm as described previously [7].

### Late phase allergic reactions

Four weeks after the transplantation of BMMCs to W/W^v^ mice, the mice were sensitized with anti-DNP IgE (20 µg/mice) intravenously. Twenty-four hours later, mice were challenged with epicutaneous application of 10 µl of DNFB (0.2% wt/vol) in acetone/olive oil (4∶1) to both sides of the ears. Ear swelling was assessed before (base line) and 2, 4, 6, 8, 12, 24, and 48 hours after the challenge.

### IgE receptor engagement

For stimulation of BMMCs via Fcε receptors, cells were incubated with mouse anti-trinitrophenol (TNP) IgE (1 µg/ml, BD Biosciences) at 37°C for 2 hours, washed twice with PBS, and then incubated with TNP^−34^-BSA (50 ng/ml, Bioresearch Technologies, Novato, CA) at 37°C for the indicated times in Tyrode's buffer (130 mmol/L NaCl, 5 mmol/L KCl, 1.4 mmol/L CaCl2, 1 mmol/L MgCl2, 5.6 mmol/L glucose, 10 mmol/L HEPES, and 0.1% BSA, PH 7.4). In some experiments, BMMCs were incubated with selective IKK2 inhibitor ML120B (10 µM) [16] or vehicle (0.01% DMSO) for 1 hour prior to stimulation. For inhibition of glycolysis, BMMCs were incubated with 2-DG (4.5 mg/ml) for 2 hours prior to stimulation. For inhibition of O-GlcNAcylation, BMMCs were incubated with streptozotocin (STZ; 5 mM) for 3 hours prior to stimulation.

### β-hexosaminidase assay

Enzyme activity of β-hexosaminidase was evaluated for both the supernatant and the cell lysate using p-nitrophenyl-N-acetyl β-D-glucosamine (Sigma) as a substrate. The percentage of specific β-hexosaminidase release was expressed as 100 x supernatant activity/(supernatant activity + cell lysate activity) as described previously [7].

### Measurement of cytokines

BMMCs were cultured and stimulated with IgE receptor engagement as described above. The amounts of TNF-α and IL-6 in the culture supernatant were measured by TNF-α and IL-6 ELISA kit (BD Biosciences). The assay was performed in duplicate according to the manufacturer's instruction. The detection limit was 15 pg/ml for both cytokines.

### Intracellular staining of p53

BMMCs were stimulated with IgE receptor engagement or Nutlin-3a (10 µM) for the indicated times. Cells were fixed, permeabilized with Perm/Wash buffer (BD Biosciences), stained with anti-p53-Alexa Fluor 647 (1C12, Cell Signaling Technology, Denvers, MA), and analyzed by flow cytometry.

### Annexin-V staining

To detect membrane fusion of BMMCs, cells were stained with Annexin-V-PE (BD Biosciences) in Annexin-V binding buffer (BD Biosciences) for 15 minutes and analyzed by flow cytometry.

### Reporter gene assay

Expression vectors for p65 [17], IKK2SE [7], and p53 [18] and NF-κB-reporter-construct [19] were described previously. HEK293 cells were transiently transfected with these plasmids using Effectene transfection reagent (Qiagen, Valencia, CA). Cells were lysed and relative light units were assessed with a dual luciferase assay system (Promega Biotech Inc., Madison, WI). Firefly luciferase activity of reporter constructs was normalized to renilla luciferase activity of pRL-TK.

### RNA purification and quantitative PCR (Q-PCR)

BMMCs were stimulated with IgE receptor engagement as described above. Total RNA was extracted using TRIzol reagent (Invitrogen, Carlsbad, CA) and reverse-transcribed into cDNA using Super Script First-Strand Synthesis System for RT-PCR (Invitrogen). Q-PCR was performed by ABI Prism 7700 sequence detection system with SYBR Green PCR master mix (Applied Biosystems, Foster City, CA) using following primer pairs:

p21^WAF1/CIP1^ (sense) 5′-CCTGGTGATGTCCGACCTG-3′;

(antisense) 5′-CCATGAGCGCATCGCAATC-3′;

BAX (sense) 5′-TGAAGACAGGGGCCTTTTTG-3′;

(antisense) 5′-AATTCGCCGGAGACACTCG-3′;

TIGAR (sense) 5′-CGCTTCGCCTTGACCGTTAT-3′;

(antisense) 5′-ACCCAGTCTCCGAAAGGGG-3′;

TNF-α (sense) 5′-ACAGAAAGCATGATCCGCG-3′;

(antisense) 5′-GCCCCCCATCTTTTGGG-3′;

IL-6 (sense) 5′-ACAACCACGGCCTTCCCTACTT-3′;

(antisense) 5′-CACGATTTCCCAGAGAACATGTG-3′;

Ifit2(sense) 5′-AGTACAACGAGTAAGGAGTCACT-3′;

(antisense) 5′-AGGCCAGTATGTTGCACACATGG-3′;

Ifit3 (sense) 5′-AGTGAGGTCAACCGGGAATCT-3′;

(antisense) 5′-TCTAGGTGCTTTATGTAGGCCA-3′;

The levels of each gene were normalized to the levels of cyclophylin A.

### Immunoprecipitation and immunoblotting

Whole cell extracts were prepared and immunoblotting was performed as described previously [20] using antisera to IKK2 (Cell Signaling Technology, Inc.), SNAP-23 (Novus Biologicals, Littleton, CO), O-GlcNAc (Covance, Princeton, NJ), p53 (Cell Signaling Technology, Inc.), and tubulin (Santa Cruz Biotechnology, Inc. Santa Cruz, CA). Antiserum to phospho-SNAP-23-Ser95 was a kind gift from Dr. Roche (National Institute of Health, Bethesda, MD). The specificity of these antibodies was described previously [21]. Immunoprecipitations were performed using anti-IKK2 antibodies (Cell Signaling Technology, Inc.) with Protein G Sepharose (Amersham Biosciences, Sweden). The aliquot of lysates was subjected to immunoblotting.

### Data analysis

Data are summarized as means ± SD. The statistical analysis of the results was performed by the unpaired t-test. p values <0.05 were considered significant.

## Results

### p53 expression is enhanced in mast cells upon IgE-mediated stimulation

**Figure 1:**
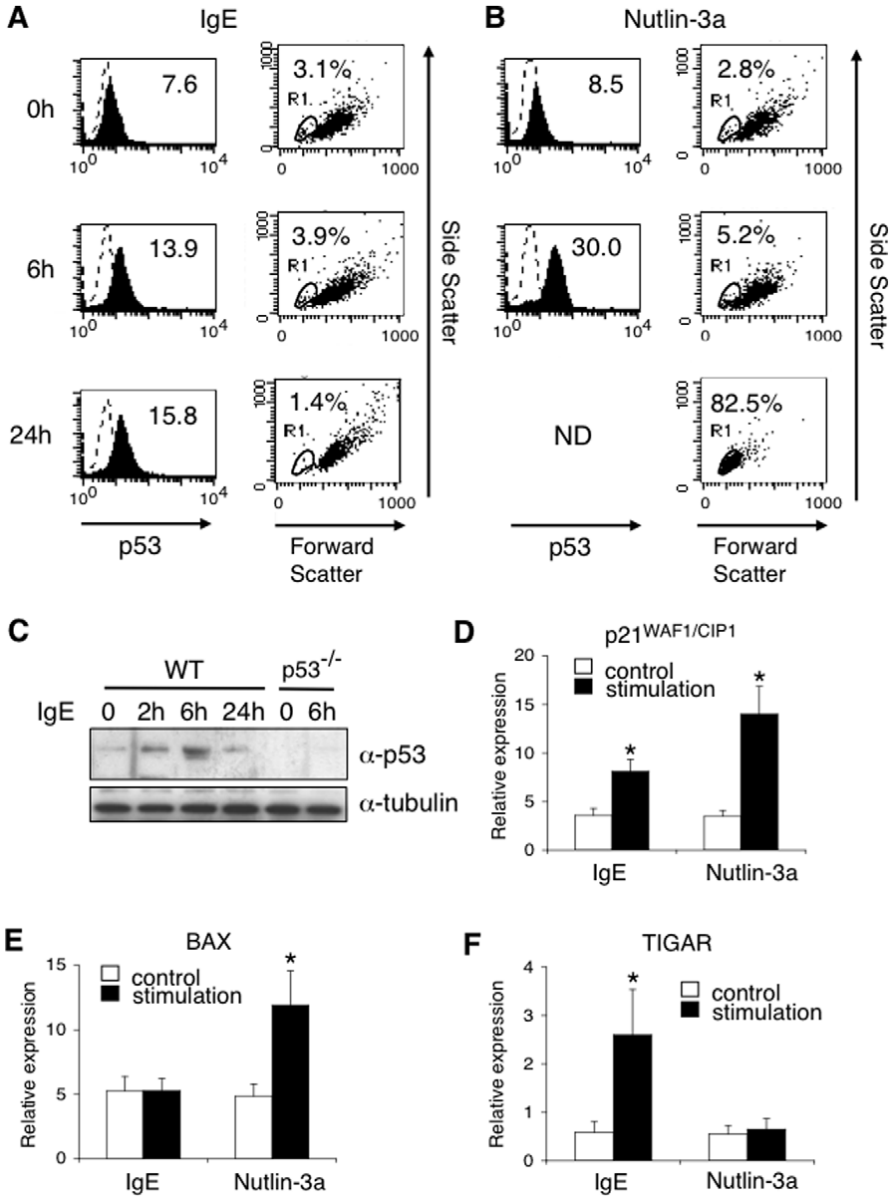
Upregulation of p53 expression upon IgE-mediated stimulation. (**A–B**) BMMCs were stimulated with IgE receptor engagement (**A**) or Nutlin-3a (10 µM) (**B**) for the indicated times as described in the **Methods**. Cells were fixed, permeabilized, incubated with anti-p53-Alexa Fluor 647 (filled histograms) or isotype-matched IgG (dot line), and analyzed by flow cytometry. p53 expression at 24 hours after Nutlin-3a stimulation could not be analyzed because of apoptosis (ND; not determined). Apoptotic cells were assessed by dot plot analysis with Forward/Side Scattering analysis (Gate R1). Shown are representative data from 5 independent experiments. (**C**) WT BMMCs or p53^−/−^ BMMCs were stimulated with IgE receptor engagement as described in the **Methods**. Before and 2, 6, and 24 hours after IgE receptor engagement, the expression of p53 and tubulin (as a control) was analyzed by immunoblotting. Shown are representative blots from 4 independent experiments. (**D–F**) BMMCs were stimulated with (black columns) or without (white columns) either IgE receptor engagement or Nutlin-3a. Four hours later, total RNA was isolated and Q-PCR analysis was performed. The expression of p21^WAF1/CIP1^ (**D**), BAX (**E**), or TIGAR (**F**) was normalized to the expression of cyclophylin A. Data are means ± SD of relative expression from 5 independent experiments. *significantly different from the mean value of controls, *p<0.01.

To determine the role of p53 in antigen receptor-mediated stimulation in mast cells, we first examined the kinetics of p53 expression upon IgE-mediated stimulation in bone marrow-derived mast cells (BMMCs). As shown in left panels of Figure 1A, IgE-mediated stimulation increased protein levels of p53 in BMMCs. Notably, the majority of BMMCs did not undergo apoptosis (Figure 1A, right panels) in spite of an increase in p53 expression. In contrast, Nutlin-3a [22], a compound that induces p53 expression by blocking the interaction between p53 and Mdm2 [23], strongly increased protein levels of p53 (Figure 1B, left panels) and induced apoptosis in mast cells (Figure 1B, right panels). Western blotting also showed that IgE-mediated stimulation increased the expression of p53 in wild type (WT) BMMCs but not in p53-deficient (p53^−/−^) BMMCs (Figure 1C). Not only Nutlin-3a but also IgE-mediated stimulation induced mRNA expression of p21^WAF1/CIP1^, one of direct targets of p53 [24], in BMMCs (Figure 1D). Importantly, whereas Nutlin-3a induced the expression of BAX, a pro-apoptotic target of p53 [25], but not of TIGAR, an anti-apoptotic target of p53 [25], in BMMCs, IgE-mediated stimulation induced the expression of TIGAR but not of BAX in BMMCs (Figure 1E–F). PUMA, another pro-apoptotic target of p53 [26], was not induced by IgE-mediated stimulation or Nutlin-3a (data not shown). These results suggest that IgE-mediated stimulation induces p53 expression without the induction of apoptosis in mast cells, presumably because of the induction of anti-apoptotic targets rather than pro-apoptotic targets.

### p53 expression is not required for the development of mast cells

We next examined whether the development of mast cells is affected by the lack of p53 using p53^−/−^ mice. The numbers of mast cells in the peritoneal cavity (Figure S1A), skin dermis (ear) (Figure S1B), or jejunal mucosa (Figure S1C) were indistinguishable between WT mice and p53^−/−^ mice. The development of BMMCs in p53^−/−^ mice was also similar to that in WT mice and more than 98% of cells recovered after 4 weeks of culture were c-kit positive (data not shown). The expression levels of c-kit and FcεRIα (Figure S2A) as well as morphological features (Figure S2B) were indistinguishable between WT and p53^−/−^ BMMCs. The lack of p53 expression in BMMCs from p53^−/−^ mice was confirmed by flow cytometry (Figure S2C). These results indicate that p53 is not required for the development of mast cells.

### Lack of p53 in mast cells results in enhanced anaphylaxis and degranulation

**Figure 2:**
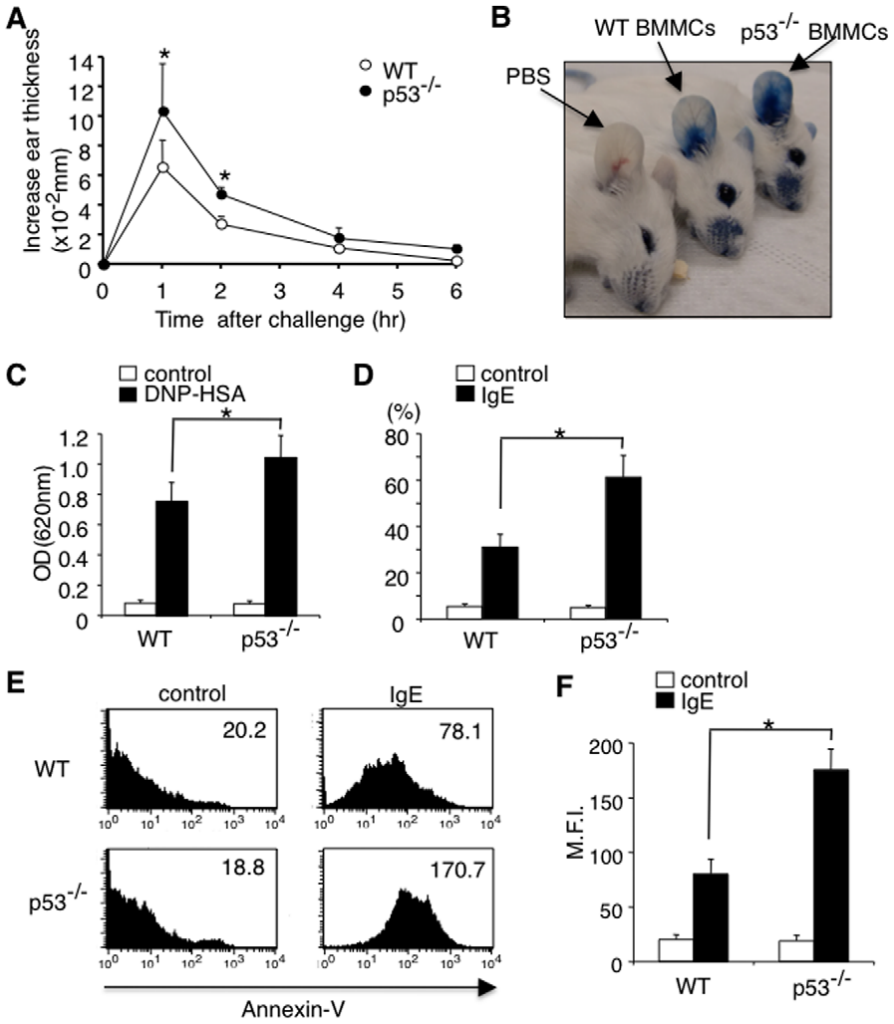
Lack of p53 expression in mast cells results in enhanced anaphylaxis and degranulation. (**A–C**) WT BMMCs or p53^−/−^ BMMCs were injected intradermally into the right ear of WBB6F1-W/W^v^ mice (W/W^v^ mice). Four weeks later, passive cutaneous anaphylaxis (PCA) was induced as described in the **Methods**. Ear swelling was quantified before (base line) and 1, 2, 4, and 6 hours after antigen challenge. n = 6 for each group. *significantly different from the mean value of WT BMMCs, *p<0.01. (**B**) Representative photograph of leakage of injected Evans blue (EB) dye in the ear in W/W^v^ mice reconstituted with p53^−/−^ BMMCs or WT BMMCs. (**C**) Quantification of extravasated EB dye in the ear specimens. Data are means ± SD of absorbance at 620 nm, n = 6, each, *p<0.01. (**D**) WT BMMCs or p53^−/−^ BMMCs were stimulated with (black columns) or without (white columns) IgE receptor engagement. Degranulation was assessed by β-hexosaminidase assay. Data are means ± SD of the percent β-hexosaminidase release, n = 6, *p<0.01. (**E, F**) WT BMMCs or p53^−/−^ BMMCs were stimulated with or without IgE receptor engagement for 1 hour. Membrane fusion of BMMCs was assessed by Annexin-V binding by using flow cytometry. Shown are the representative histograms of Annexin-V binding (**E**) and means ± SD of mean fluorescence intensity (M.F.I.) of Annexin-V binding (**F**). n = 5, each, *p<0.01.

To assess the physiological significance of p53 up-regulation upon IgE-mediated stimulation, we investigated the role of p53 in IgE-mediated anaphylaxis using a mast cell knock-in system [1], [2]. In this experiment, cultured p53^−/−^ BMMCs or WT BMMCs were intradermally transplanted into mast cell-deficient WBB6F1-Kit^w^/Kit^w-v^ (W/W^v^) mice. Four weeks after the transplantation, these mice were sensitized with DNP-specific IgE antibody and then challenged with DNP-HSA. Importantly, W/W^v^ mice reconstituted with p53^−/−^ BMMCs exhibited enhanced ear thickness (Figure 2A) and enhanced leakage of Evans blue dye (Figure 2B–C) as compared to W/W^v^ mice reconstituted with WT BMMCs. On the other hand, the numbers of mast cells in the ear were nearly identical between W/W^v^ mice reconstituted with p53^−/−^ BMMCs and WT BMMCs (Figure S3). Therefore, it is suggested that the enhanced anaphylaxis in W/W^v^ mice reconstituted with p53^−/−^ BMMCs is due to hyper-reactivity of p53^−/−^ mast cells to IgE-mediated stimulation.

To address the cellular basis for the enhanced anaphylaxis in W/W^v^ mice reconstituted with p53^−/−^ BMMCs, we next compared IgE-mediated degranulation between p53^−/−^ BMMCs and WT BMMCs. Consistent with the enhanced anaphylaxis in W/W^v^ mice reconstituted with p53^−/−^ BMMCs, p53^−/−^ BMMCs showed a clear increase in IgE-induced degranulation assessed by the release of β-hexosaminidase (Figure 2D). p53^−/−^ BMMCs also showed an enhanced binding to Annexin-V, which specifically binds to phosphatidylserine that is exposed to outer surface by degranulation in mast cells, as compared to WT BMMCs (Figure 2E–F). These results suggest that p53 attenuates IgE-induced degranulation in mast cells.

### Lack of p53 in mast cells results in enhanced kinase activity of IKK2

**Figure 3:**
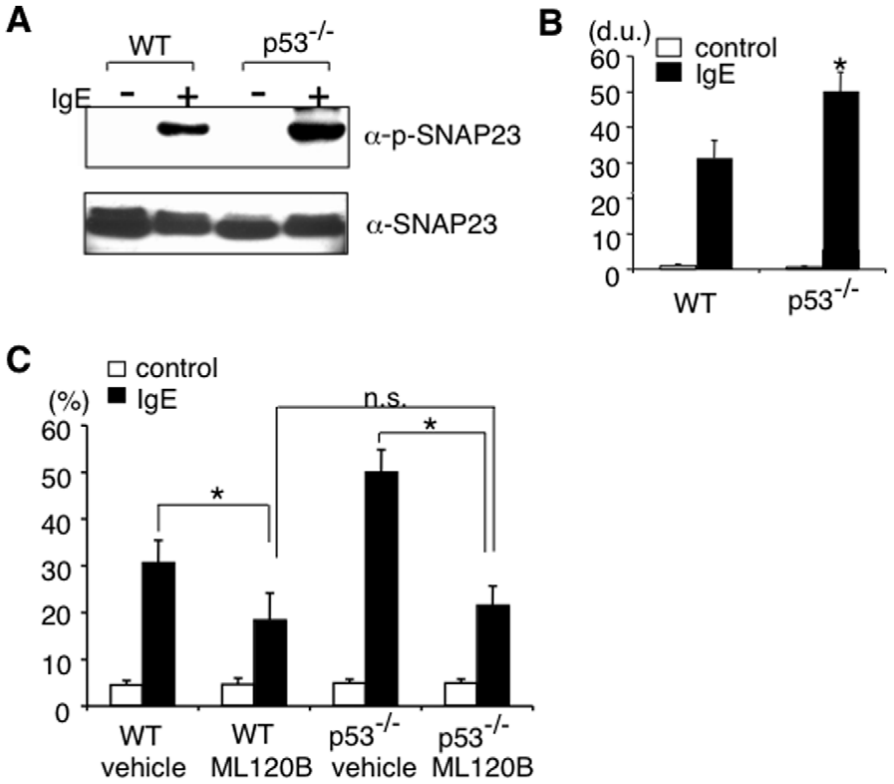
Lack of p53 expression in mast cells results in enhanced kinase activity of IKK2 and degranulation. (**A, B**) IgE-mediated SNAP-23 phosphorylation in WT and p53^−/−^ BMMCs was analyzed by immunoblotting using phosphorylation site-specific antibody. The representative blots from 4 independent experiments (**A**) and means ± SD of the density of the blots (**B**) are shown. *significantly different from the mean value of WT BMMCs, *p<0.01. d.u. = density unit. (**C**) WT and p53^−/−^ BMMCs were incubated with ML120B (10 µM) or vehicle (DMSO (0.01%)). Sixty minutes later, IgE-mediated degranulation was assessed as described in **Figure 2D**. Data are means ± SD of the percent β-hexosaminidase release, n = 5, *p<0.05. n.s. = not significant.

Given that the phosphorylation of SNAP23 by IKK2 is a key biological process in mast cell degranulation and early phase allergic reactions [7], we next examined whether p53 affects IKK2 functions. Importantly, we found a significant increase in IgE-induced phosphorylation of SNAP23 in p53^−/−^ BMMCs (Figure 3A–B). We also examined the role of IKK2 in the enhanced degranulation in p53^−/−^ BMMCs by using an IKK2-specific inhibitor ML120B [16], [27]. The efficacy of ML120B was confirmed by its effect on TNF-α-induced IκBα phosphorylation in WT BMMCs (Figure S4). Consistent with our previous report [7], ML120B inhibited IgE-induced degranulation in WT BMMCs (Figure 3C). In addition, ML120B significantly inhibited IgE-induced degranulation in p53^−/−^ BMMCs (Figure 3C) to the level similar to that in ML120B-treated WT BMMCs (Figure 3C). Taken together, these results suggest that IKK2 activation is likely to be involved in the increase of IgE-induced degranulation in p53^−/−^ BMMCs.

### IKK2 is O-GlcNAcylated in the absence of p53 in mast cells

**Figure 4:**
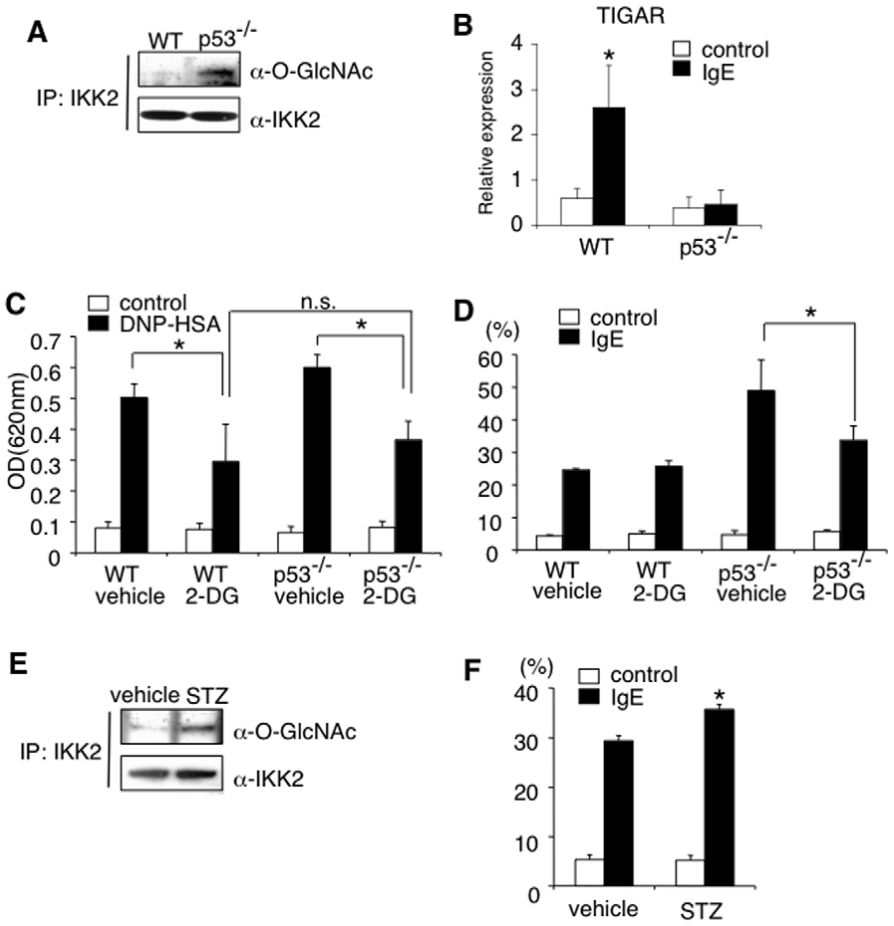
Lack of p53 expression in mast cells results in enhanced O-GlcNAcylation of IKK2. (**A**) O-GlcNAcylation of IKK2 in WT or p53^−/−^ BMMCs was detected by immunoprecipitation with anti-IKK2 antibody, followed by immunoblotting with anti-O-GlcNAc antibody. Shown are representative blots from 5 independent experiments. (**B**) The expression of TIGAR was assessed by Q-PCR analysis as described in **Figure 1F**. Data are means ± SD, n = 5, *significantly different from the mean value of controls, *p<0.01. (**C**) 2-deoxy-D-glucose (2-DG) were given to W/W^v^ mice reconstituted with WT or p53^−/−^ BMMCs. Twenty hours later, PCA reaction was assessed as described in **Figure 2C**. Data are means ± SD, n = 6 for each, *p<0.05. (**D**) WT or p53^−/−^ BMMCs were incubated with 2-DG (4.5 mg/ml) for 2 hours. IgE-mediated degranulation was assessed as described in **Figure 2D**. Data are means ± SD of the percent β-hexosaminidase release, n = 5, *p<0.01. (**E**) WT BMMCs were incubated with streptozotocin (STZ; 5 mM) or vehicle for 3 hours. O-GlcNAcylation of IKK2 was detected as described in **Figure 4A**. Shown are representative blots from 5 independent experiments. (**F**) After incubation with STZ for 3 hours, WT BMMCs were subjected to IgE-mediated degranulation. Data are means ± SD of the percent β-hexosaminidase release, n = 5, *significantly different from the mean value of vehicle, *p<0.05.

Recently, it has been reported that the absence of p53 results in the enhanced catalytic activity of IKK2 by causing O-GlcNAcylation at Ser733 in fibroblasts [28], [29]. We therefore examined O-GlcNAcylation of IKK2 in p53^−/−^ BMMCs and found that IKK2 was indeed O-GlcNAcylated in p53^−/−^ BMMCs but not in WT BMMCs (Figure 4A). In addition, the induction of TIGAR, which functions as a potent inhibitor of glycolysis [25], was significantly increased in WT BMMCs but not in p53^−/−^ BMMCs upon IgE-mediated stimulation (Figure 4B). Therefore, it is possible that p53 reduces the levels of O-GlcNAcylation of IKK2 via the induction of TIGAR.

To determine whether O-GlcNAcylation of IKK2 in p53^−/−^ BMMCs is involved in the enhanced anaphylaxis in W/W^v^ mice reconstituted with p53^−/−^ BMMCs (Figure 2), we investigated the effect of 2-deoxy-D-glucose (2-DG), a glycolytic inhibitor, on IgE-mediated anaphylaxis. As shown in Figure 4C, 2-DG inhibited IgE-induced leakage of Evans blue dye not only in W/W^v^ mice reconstituted with WT BMMCs but also in W/W^v^ mice reconstituted with p53^−/−^ BMMCs. 2-DG also inhibited IgE-induced degranulation of p53^−/−^ BMMCs (Figure 4D). Because O-GlcNAcylation of IKK2 has been shown to enhance its kinase activity [29], we next examined the effect of streptozotocin (STZ), an O-GlcNAcase inhibitor, on IgE-induced degranulation. STZ enhanced O-GlcNAcylation of IKK2 (Figure 4E) and enhanced IgE-induced degranulation (Figure 4F) in WT BMMCs. Taken together, these results suggest that O-GlcNAcylation of IKK2 is a possible mechanism of the enhanced degranulation in p53^−/−^ BMMCs.

### p53 attenuates late phase allergic reactions and NF-κB-mediated cytokine production

**Figure 5:**
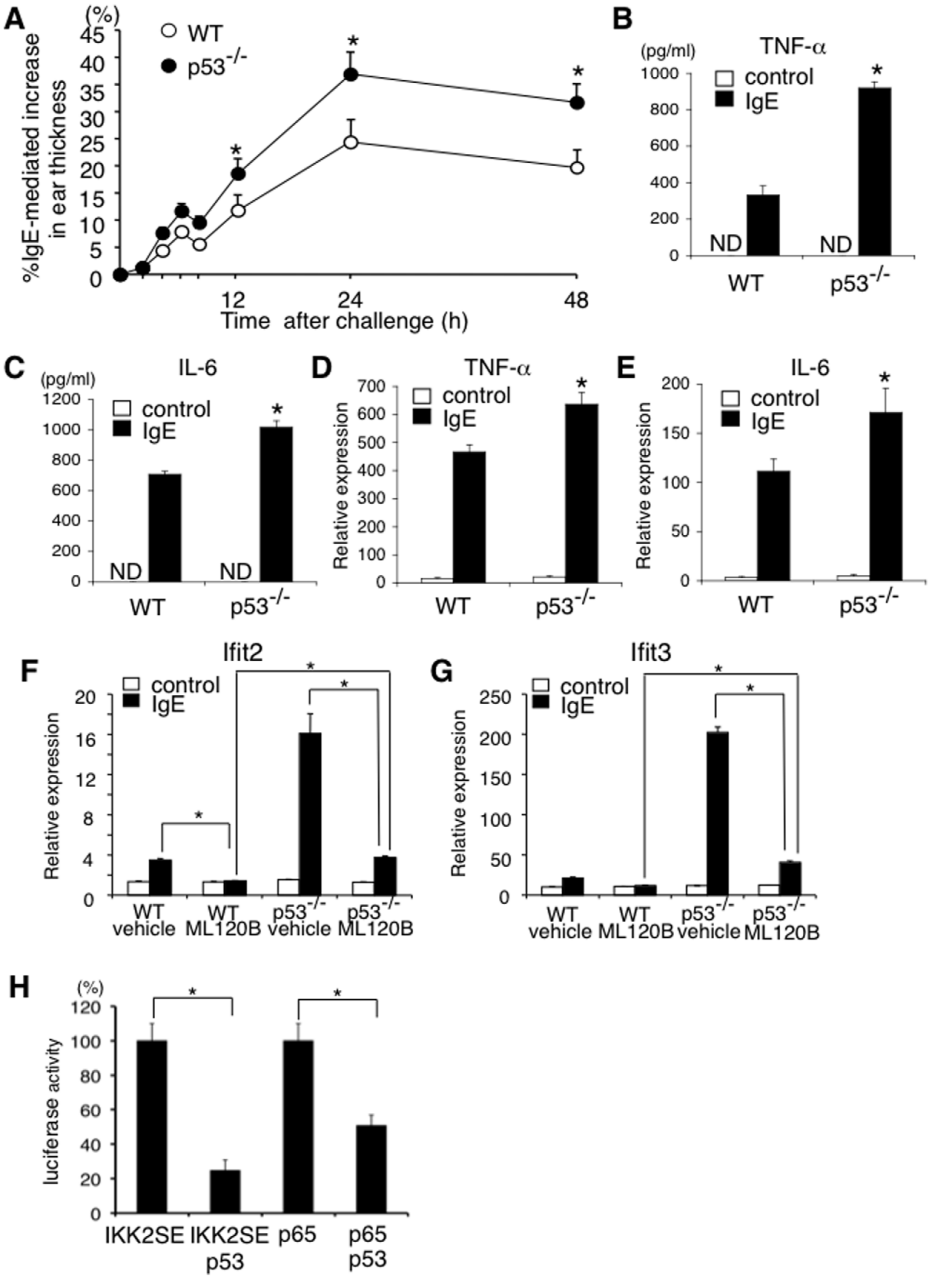
p53 attenuates late phase allergic reactions and NF-κB-mediated cytokine production. (**A**) W/W^v^ mice were reconstituted with WT or p53^−/−^ BMMCs and late phase allergic reactions were induced as described in the **Methods**. Data were means ± SD of IgE-mediated increase in ear thickness, n = 6 for each genotype, *significantly different from the mean value of WT BMMCs, *p<0.01. (**B–E**) WT or p53^−/−^ BMMCs were stimulated with (black columns) or without (white columns) IgE receptor engagement. (**B, C**) The levels of TNF-α and IL-6 in the supernatants were quantified by ELISA. Data were means ± SD, n = 5, *significantly different from the mean value of WT BMMCs, *p<0.01. ND = not detected. (**D, E**) Two hours after IgE receptor engagement, the levels of TNF-α and IL-6 mRNA were quantified by Q-PCR. Data were means ± SD of relative expression, n = 5, *significantly different from the mean value of WT BMMCs, *p<0.05. (**F, G**) WT or p53^−/−^ BMMCs were incubated with ML120B or vehicle for 60 minutes and then stimulated with (black columns) or without (white columns) IgE receptor engagement. Sixty minutes later, the expression of Ifit2 (**F**) and Ifit3 (**G**) was assessed by Q-PCR analysis. n = 5, *p<0.01. (**H**) HEK293 cells were transfected with the expression vectors of IKK2SE, p65, and/or p53 in the presence of NF-κB-luciferase reporter construct. The luciferase activity of NF-κB reporter construct was quantified as described in the **Methods.** Data are means ± SD of the percent luciferase activity, n = 5, *p<0.01.

Late phase responses in IgE-mediated anaphylaxis are promoted by mast cell-derived pro-inflammatory cytokines such as TNF-α and IL-6 [1]. We next examined late phase responses in IgE-mediated anaphylaxis in W/W^v^ mice reconstituted with p53^−/−^ BMMCs and WT BMMCs. As shown in Figure 5A, W/W^v^ mice reconstituted with p53^−/−^ BMMCs exhibited strong ear swelling as compared with W/W^v^ mice reconstituted with WT BMMCs. Consistent with strong ear swelling in W/W^v^ mice reconstituted with p53^−/−^ BMMCs, IgE-induced TNF-α and IL-6 secretion was significantly facilitated in p53^−/−^ BMMCs as compared with that in WT BMMCs (Figure 5B–C). IgE-induced mRNA induction of TNF-α and IL-6 was also facilitated in p53^−/−^ BMMCs (Figure 5D–E). These results suggest that p53 inhibits IgE-induced pro-inflammatory cytokine production in mast cells and inhibits subsequent late phase responses in IgE-mediated anaphylaxis.

In the NF-κB pathways, p65 directly induces the expression of a number of target genes in a various cell types [3]. We next examined IgE-induced expression of Ifit2 and Ifit3, whose induction by LPS is shown to be dependent on p65 [30], in WT BMMCs and p65-deficient (p65^−/−^) BMMCs. As expected, the induction of Ifit2 and Ifit3 by IgE-mediated stimulation was detected in WT BMMCs but not in p65^−/−^ BMMCs (Figure S5A–B), indicating that IgE-mediated induction of Ifit2 and Ifit3 is also dependent on p65. IKK2-specific inhibitor ML120B also inhibited IgE-mediated induction of Ifit2 and Ifit3 in WT BMMCs (Figure 5F–G). Consistent with the enhanced catalytic activity of IKK2 in p53^−/−^ BMMCs (Figure 3A–B), IgE-mediated induction of Ifit2 and Ifit3 was significantly enhanced in p53^−/−^ BMMCs and ML120B inhibited IgE-mediated induction of Ifit2 and Ifit3 in p53^−/−^ BMMCs (Figure 5F–G). However, importantly, even in the presence of ML120B, IgE-mediated induction of Ifit2 and Ifit3 was still observed in p53^−/−^ BMMCs (Figure 5F–G). The residual activity of p65-dependent transcription in ML120B-treated p53^−/−^ BMMCs but not in ML120B-treated WT BMMCs suggest that besides the inhibition of IKK2, p53 may directly regulate the activity of p65.

To address the possibility that p53 directly inhibits the activity of p65, we finally investigated the effect of ectopic expression of p53 on NF-κB-dependent promoter activation induced by a phosphomimetic mutant IKK2, IKK2SE, or by p65. As shown in Figure 5H, p53 repressed not only IKK2SE-induced activation of NF-κB-dependent promoter but also p65-induced activation of NF-κB-dependent promoter, indicating that p53 directly represses p65 activity. Taken together, these results indicate that p53 attenuates IgE-mediated expression of pro-inflammatory cytokine genes not only by suppressing the catalytic activity of IKK2 through the modulation of glycosylation but also by suppressing the transcriptional activity of p65.

## Discussion

In this study, we show that p53 functions as a negative regulator of mast cell activation through the inhibition of NF-κB pathways. We found that protein levels of p53 were up-regulated upon IgE-mediated stimulation in mast cells without the induction of apoptosis (Figure 1). We also found that the lack of p53 in mast cells results in enhanced IgE-induced degranulation (Figure 2) and cytokine production (Figure 5), leading to enhanced responses in both early phase (Figure 2) and late phase (Figure 5) of IgE-mediated anaphylaxis in vivo. These results suggest that p53 is involved in negative feedback regulation of IgE-madiated mast cell activation.

It is well established that stress-associated stimulation, such as apoptosis, cell cycle arrest, and senescence, increases p53 expression, leading to apoptosis of various cell types [9]–[12]. In contrast, we found that IgE-induced increase of p53 expression did not affect mast cell survival (Figure 1A). We also found that whereas Nutlin-3a, a compound that induces p53 expression [23], induced the expression of a pro-apoptotic p53 target BAX but not of an anti-apoptotic p53 target TIGAR in mast cells, IgE-mediated stimulation induced the expression of TIGAR but not of BAX in mast cells (Figure 1E–F). These results suggest that the balance between a pro-apoptotic p53 target gene and an anti-apoptotic p53 target gene could be involved in the non-apoptotic feature of mast cells upon IgE-induced p53 expression.

The reasons why IgE-mediated stimulation induces an anti-apoptotic p53 target TIGRR while p53 activation by Nutlin-3a induces a pro-apoptotic p53 target BAX in mast cells are still unclear. It is well established that Nutlin-3a induces p53 expression by blocking the interaction between p53 and Mdm2 [23]. On the other hand, although a molecular machinery_of IgE-mediated up-regulation of p53 has not been clarified, IgE-mediated stimulation activates several signaling pathways including PKC pathway, calcineurin/NFAT pathway, Ras/RAF/MEK/ERK pathway, Rac/MEKK/JNK pathway, and IKK complex/NF-κB pathway in mast cells [1]. Therefore, it is possible that the activation of these signaling pathways by IgE-mediated stimulation affects the balance between the expression of BAX and TIGAR in mast cells.

Regarding the mechanism by which p53 attenuates IgE-mediated stimulation, we show that the lack of p53 results in a significant increase in IgE-induced phosphorylation of SNAP23 in mast cells (Figure 3A–B), a key biological process in mast cell degranulation and early phase allergic reactions [7]. In addition, we found that the inhibition of IKK2, which phosphorylates SNAP23 upon IgE-mediated stimulation in mast cells [7], cancelled the enhanced IgE-induced degranulation in p53^−/−^ BMMCs (Figure 3C), suggesting that IKK2-mediated phosphorylation of SNAP23 is involved in the enhanced IgE-induced degranulation in p53^−/−^ mast cells. Moreover, the expression of TIGAR, which functions as a potent inhibitor of glycolysis [25], was significantly increased in WT BMMCs but not in p53^−/−^ BMMCs upon IgE-mediated stimulation (Figure 4B). Furthermore, Kawauchi et al. have recently shown that the absence of p53 results in the enhanced catalytic activity of IKK2 by causing O-GlcNAcylation in fibroblasts [29]. Taken together, these results raise the possibility that IgE-mediated stimulation induces the expression of TIGAR through the induction of p53 and subsequently TIGAR reduces the levels of O-GlcNAcylated IKK2, leading to the attenuation of IgE-mediated degranulation and cytokine production in mast cells.

We found that O-GlcNAcylation of IKK2 was detected in p53^−/−^ BMMCs but not in WT BMMCs (Figure 4A). We also found that 2-DG inhibited IgE-induced degranulation in p53^−/−^ BMMCs but not in WT BMMCs (Figure 4D). These results suggest that O-GlcNAcylation of IKK2 is almost completely inhibited by the presence of p53 in WT BMMCs. In contrast to these in vitro studies, 2-DG treatment inhibited IgE-mediated leakage of Evans blue dye in W/W^v^ mice reconstituted not only with p53^−/−^ BMMCs but with WT BMMCs (Figure 4C). The discrepancy raises the possibility that the inhibition of O-GlcNAcylation of IKK2 by p53 is incomplete in mast cells in the in vivo studies. It is also possible that the glycolytic modification of some signaling molecule besides IKK2 is involved in IgE-mediated increase_of vascular permeability in the in vivo studies. Further experiments are required to address these possibilities.

In conclusion, the evidence provided here suggests that p53 plays a crucial role in controlling the intensity and/or duration of mast cell responses to antigens by suppressing IgE receptor-IKK2-NF-κB axis at multiple steps.

## Supporting Information

Figure S1 **The numbers of mast cells in the tissues are indistinguishable between WT mice and p53^−/−^ mice.** The numbers of mast cells in the peritoneal cavity (**A**), skin dermis (ear) (**B**), and jejunal mucosa (**C**) were evaluated as described in the **Methods.** Data are means ± SD from 5 independent experiments. vcu = villus crypt unit.
(TIF)

Figure S2 **Development of IL-3-dependent bone marrow-derived mast cells (BMMCs) is normal in p53^−/−^ mice.** (**A**) Bone marrow cells from WT mice or p53^−/−^ mice were cultured in the presence of IL-3 for 4 weeks. Cells were stained with anti-FcεRIα FITC and anti-c-kit APC and analyzed by flow cytometry. Shown are representative FACS profiles from five independent experiments. (**B**) BMMCs stained with Wright-Giemsa solution are shown. (**C**) p53 expression in WT BMMCs and p53^−/−^ BMMCs was evaluated by flow cytometry.
(TIF)

Figure S3 **The numbers of mast cells in the ear skin dermis are indistinguishable between W/W^v^ mice reconstituted with WT BMMCs and p53^−/−^ BMMCs.** Four weeks after the transplantation of BMMCs, the numbers of mast cells in the ear skin dermis were assessed.
(TIF)

Figure S4 **Selective IKK2 inhibitor ML120B inhibits phosporylation of IκBα.** (**A, B**) BMMCs were incubated with ML120B (10 µM) or vehicle (DMSO (0.01%)) as a control for 60 minutes and then stimulated with or without TNF-α (10 ng/ml). Fifteen minutes later, cell lysates were recovered and analyzed by immunoblot analysis using phosphorylation site-specific antibodies. Representative blots from five independent experiments **(A)** and means ± SD of the density of blots **(B)** were shown. *significantly different from the mean value of vehicle, *p<0.01. d.u. = density unit.
(TIF)

Figure S5 **IgE-induced Ifit2 and Ifit3 expression is p65 dependent.** (**A, B**) WT BMMCs or p65^−/−^ BMMCs were stimulated with or without IgE receptor engagement. Two hours later, total RNA was extracted and Q-PCR for Ifit2 (**A**) or Ifit3 (**B**) was performed. Data are means ± SD of relative expression from 5 independent experiments. *significantly different from the mean value of controls, *p<0.01.
(TIF)
