# Correction: Tumor Suppressor p53 Functions as a Negative Regulator in IgE-Mediated Mast Cell Activation

**DOI:** 10.1371/journal.pone.0109474

**Published:** 2014-09-25

**Authors:** The PLOS ONE Staff

K.S. is supported in part by Grants-in Aids for Scientific Research from the Ministry of Education, Culture, Sports, Science, and Technology, the Japanese Government and by Global COE Program (Global Center for Education and Research in Immune System Regulation and Treatment), MEXT, Japan. This work was also supported by NIH grant number AI048034 to IMV. The funders had no role in study design, data collection and analysis, decision to publish, or preparation of the manuscript.
