# Supplementary figures and images for: Tumor Suppressor p53 Functions as a Negative Regulator in IgE-Mediated Mast Cell Activation
Source: PLoS One. 2011 Sep 23;6(9):e25412. doi: 10.1371/journal.pone.0025412 (PMC3179515; doi:10.1371/journal.pone.0025412)

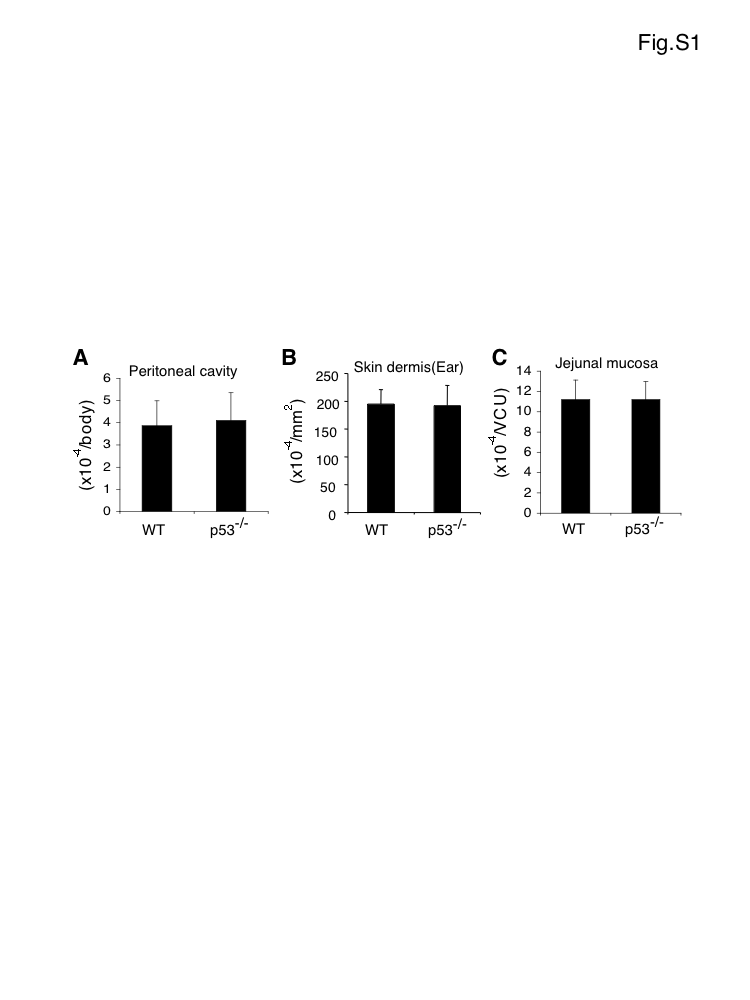

Supplement: Figure S1 — The numbers of mast cells in the tissues are indistinguishable between WT mice and p53−/− mice. The numbers of mast cells in the peritoneal cavity (A), skin dermis (ear) (B), and jejunal mucosa (C) were evaluated as described in the Methods. Data are means ± SD from 5 independent experiments. vcu = villus crypt unit. (TIF) [file pone.0025412.s001.tif]

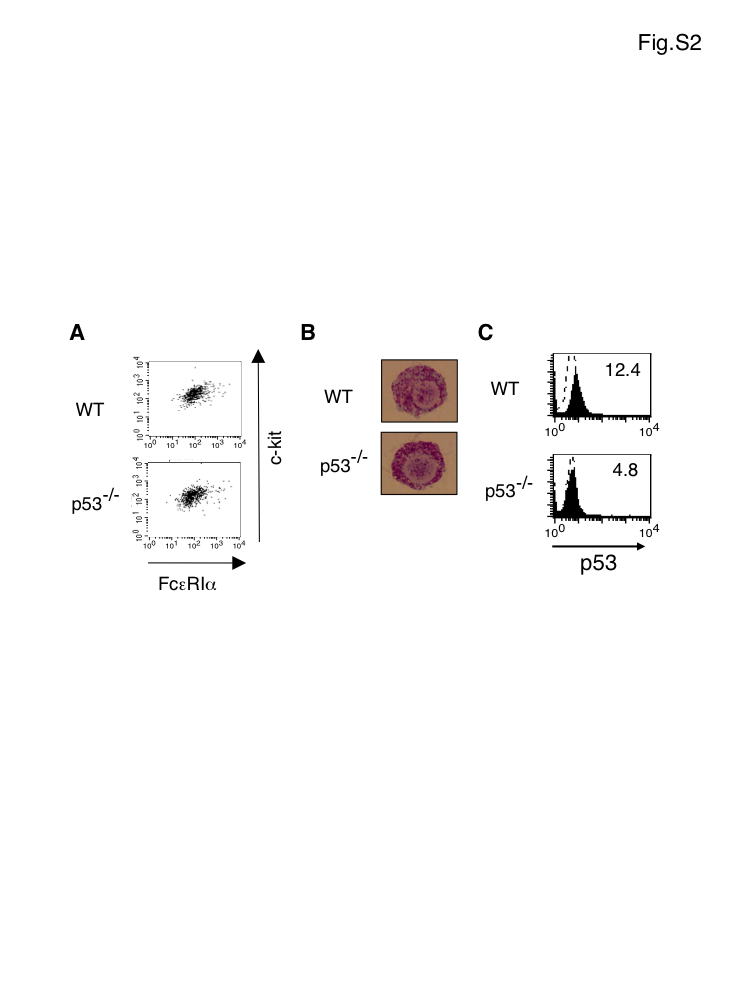

Supplement: Figure S2 — Development of IL-3-dependent bone marrow-derived mast cells (BMMCs) is normal in p53−/− mice. (A) Bone marrow cells from WT mice or p53−/− mice were cultured in the presence of IL-3 for 4 weeks. Cells were stained with anti-FcεRIα FITC and anti-c-kit APC and analyzed by flow cytometry. Shown are representative FACS profiles from five independent experiments. (B) BMMCs stained with Wright-Giemsa solution are shown. (C) p53 expression in WT BMMCs and p53−/− BMMCs was evaluated by flow cytometry. (TIF) [file pone.0025412.s002.tif]

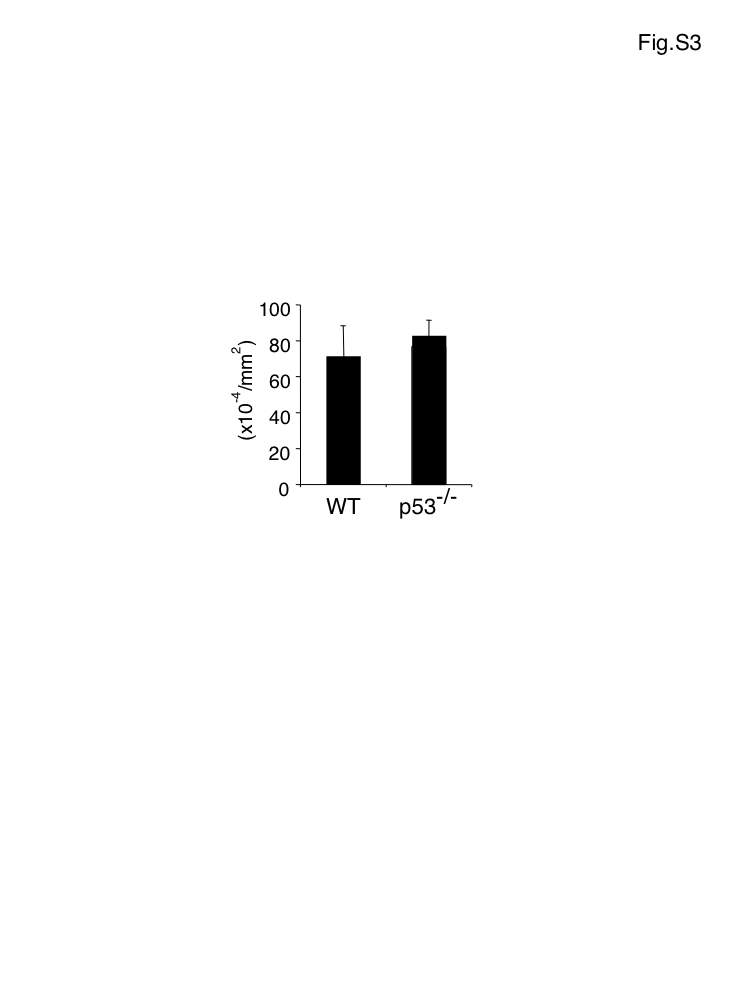

Supplement: Figure S3 — The numbers of mast cells in the ear skin dermis are indistinguishable between W/Wv mice reconstituted with WT BMMCs and p53−/− BMMCs. Four weeks after the transplantation of BMMCs, the numbers of mast cells in the ear skin dermis were assessed. (TIF) [file pone.0025412.s003.tif]

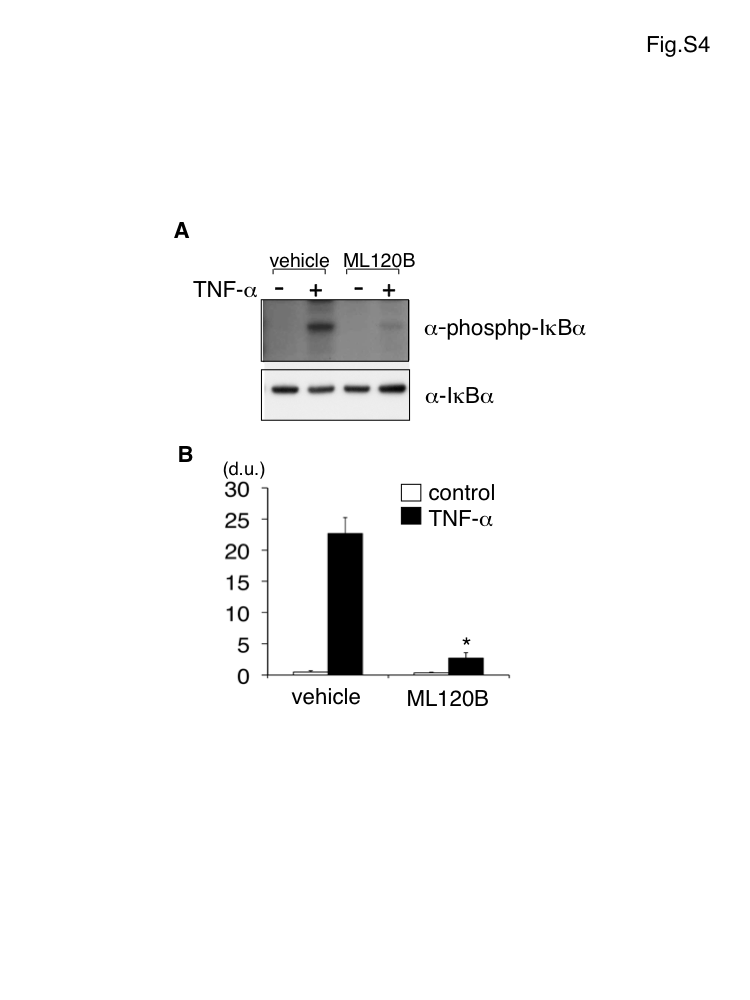

Supplement: Figure S4 — Selective IKK2 inhibitor ML120B inhibits phosporylation of IκBα. (A, B) BMMCs were incubated with ML120B (10 µM) or vehicle (DMSO (0.01%)) as a control for 60 minutes and then stimulated with or without TNF-α (10 ng/ml). Fifteen minutes later, cell lysates were recovered and analyzed by immunoblot analysis using phosphorylation site-specific antibodies. Representative blots from five independent experiments (A) and means ± SD of the density of blots (B) were shown. *significantly different from the mean value of vehicle, *p<0.01. d.u. = density unit. (TIF) [file pone.0025412.s004.tif]

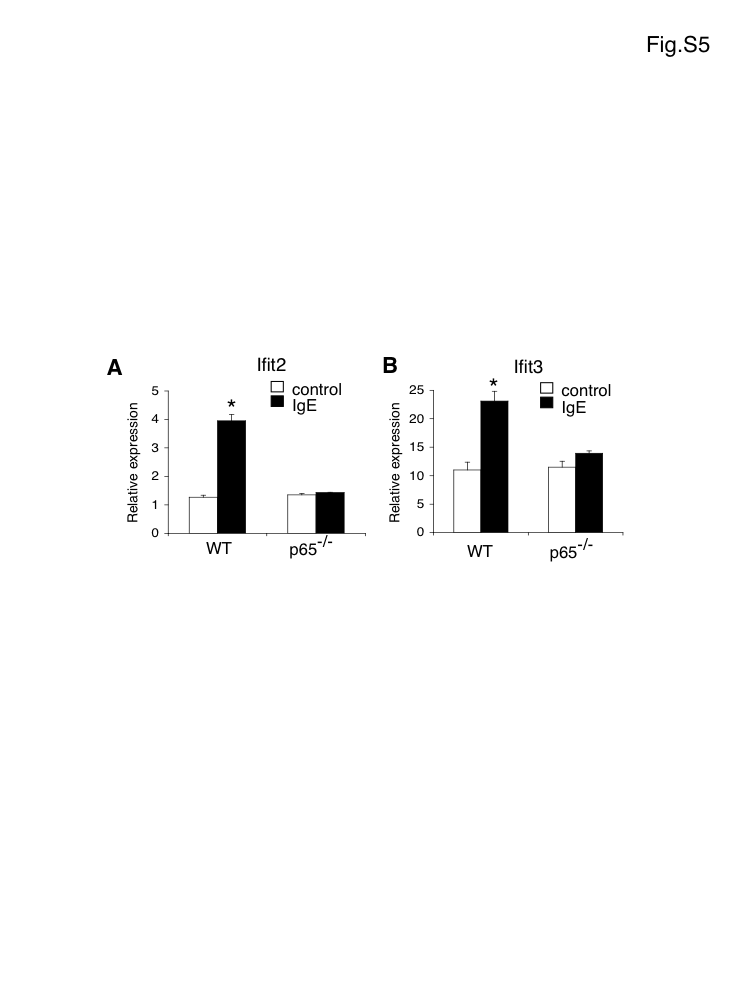

Supplement: Figure S5 — IgE-induced Ifit2 and Ifit3 expression is p65 dependent. (A, B) WT BMMCs or p65−/− BMMCs were stimulated with or without IgE receptor engagement. Two hours later, total RNA was extracted and Q-PCR for Ifit2 (A) or Ifit3 (B) was performed. Data are means ± SD of relative expression from 5 independent experiments. *significantly different from the mean value of controls, *p<0.01. (TIF) [file pone.0025412.s005.tif]
